# Socio-cultural insights and lymphatic filariasis control – lessons from the Pacific

**DOI:** 10.1186/1475-2883-6-3

**Published:** 2007-02-17

**Authors:** Shona Wynd, David N Durrheim, Jaime Carron, Billy Selve, JP Chaine, Peter A Leggat, Wayne Melrose

**Affiliations:** 1 World Health Organization Collaborating Centre for the Control of Lymphatic Filariasis, James Cook University, Douglas, Townsville, 4811, Australia; 2 Tabubil Hospital & Community Centre, Western Province, Papua New Guinea; 3 Divine Word University, Madang, Papua New Guinea; 4 Pacific Islands Health Officers' Association, B.O. Box 2059, Pohnpei, 96941, Federated States of Micronesia

## Abstract

**Background:**

Sustainable and equitable health programmes require a grounded understanding of the context in which they are being implemented. This socio-cultural understanding is pivotal for effective delivery of elimination programmes. Standardised valid methods are needed for gathering authentic socio-cultural insights. The currently recommended protocol for collecting Lymphatic Filariasis (LF) related socio-cultural data, while moving in the right direction, is inadequate. To collect data which provides an understanding of local health beliefs and practices, and communities' understanding of LF, techniques must be developed that are both valid and time efficient. An approach developed in the Pacific provides a basic snapshot of socio-cultural insights which are crucial to the development of relevant and sustainable health education and elimination programmes.

**Summary:**

The increasing interest in socio-cultural LF research presents a unique opportunity for coupling socio-cultural and bio-medical understandings of LF. To address the backlog in the socio-cultural sphere will require investment of time and effort to integrate valid qualitative approaches into current data collection methodologies.

## Background

Lymphatic filariasis (LF) exacts an enormous infectious toll, affecting approximately 80 tropical and sub-tropical countries. Prevalence mapping is ongoing but a billion people are estimated to be at risk of infection, with 120 million already infected and 40 million seriously incapacitated or disfigured by the disease [1]. While the disease does not result directly in death, it is, after mental illness, the second most common cause of long-term disability globally [2]. One third of people infected with LF live in India, a third live in Africa and the remainder live in Papua New Guinea, Southeast Asia, the Pacific Islands and the Americas.

Limited sociological data has been gathered to illuminate the sociocultural factors that influence lymphatic filariasis (LF) occurrence and the success of programmes implemented to eliminate the disease. While there is a growing recognition of the necessity of socio-cultural insights to support effective elimination programmes [3], there remains a dearth of field expertise and a lack of pragmatic data collection techniques to fill the void.

We highlight the important role that socio-cultural insights play in supporting an effective elimination programme. Further we introduce a useful methodology for surveying the knowledge and perceptions of local populations and briefly illustrate its value following application in two Pacific contexts.

Papua New Guinea (PNG) is the country with the greatest remaining burden of lymphatic filariasis (LF) in the Western Pacific Region [4]. Although the current National Health Plan has integrated LF planning into its vector-borne disease control programme [5], PNG is the only endemic country in the Region that has not yet introduced a countrywide programme to eliminate LF.

However, there have been some government-private sector initiatives, such as that on Misima Island in rural Milne Bay Province, Papua New Guinea, where the government and private sector have collaborated to implement a pilot control programme [6]. Historically, Misima Island has experienced a high prevalence of filariasis. A survey conducted on the Island in 1997 found a prevalence rate of 56% using immunochromatographic card tests [7].

Over a five year period the Misima Island LF control programme combined ongoing awareness campaigns with annual single-dose administration of diethylcarbamazine (DEC) and albendazole for all community members. This approach dramatically reduced infection rates to below two percent [7]. While the success of the Misima Island programme has been attributed to the utilisation of local social structures and communication networks during the mass drug administration (MDA) campaigns, little is known about the perceptions of Misima Island residents about LF and the annual MDA.

In contrast to PNG, the Federated States of Micronesia (FSM), consisting of the states of Yap, Chuuk, Pohnpei and Kosrae, was until recently thought to have a very low incidence of LF and not considered endemic [4]. There was very little understanding of socio-cultural beliefs regarding LF or health-seeking behaviour. Surveys using immunochromatographic card tests conducted in 1999/2000 and 2001 indicated that Chuuk Lagoon Islands and Yap State had incidences of 0.2% and 0.5%, respectively. However, in 2002 a survey found a 34.4% prevalence rate by rapid card tests and a microfiliaremia rate of 18.7% on the island of Satawal in Chuuk [8]. In response an MDA was conducted in 2003 and a second is planned for the end of 2004.

## Discussion

### Recognising the value of socio-cultural insights

While explicit acknowledgement by the wider LF research and control community of the importance of socio-behavioural understandings for achieving optimal programme success is encouraging [9], the WHO recommended approach for gathering socio-cultural information from affected communities had major shortcomings [10]. The protocol reflects a philosophy that aims to capture superficial data from a wide geographical catchment area, focusing on a limited number of data items collected from a small number of respondents in a large number of sites. The design attempts to move towards the qualitative paradigm, but fails to make sufficient commitment to pursuing new understanding from the point of view of the local communities. It overlooks the fact that local definitions and explanations for LF are completely unrelated to scientific, bio-physical models of health. The protocol assumes a shared "western" understanding of health and of LF, and attempts to confirm whether or not communities are compliant with bio-medically oriented local elimination programmes. This resulting hybrid of questions that appeared to be qualitatively oriented, does not actually achieve the central goal of improving understanding of the degree to which communities are actually aware that LF is present, and the manner in which they respond to the illness.

We adapted the protocol in two stages. In the first stage we reduced the recommended size of geographical area covered, but surveyed more people within that area. In PNG we found that adaptation of this protocol allowed a richer understanding in a smaller sample of communities. We conducted eight focus group discussions and key informant interviews in four villages. The villages were purposively selected to take account of differential remoteness, proximity to the employing mine and available health facilities. Purposive sampling was used to identify groups of teenage males, teenage females, adult males and adult females in each village, with 137 volunteer participants. Two to four key informants per village were identified and interviewed for a total of 13 individuals. The key informants were generally prominent village members, such as ward councillors, pastors, Ward Development Committee members, teachers, elders, and Women's Fellowship leaders.

It is clear that many of the questions in the currently recommended protocol were developed for use in populations where chronic manifestations of LF remain commonplace, and this is certainly not the case on Misima Island or many other Pacific countries. If the large number of prompts related specifically to elephantiasis and hydrocoele are rigorously adhered to, little time remains to probe more subtle presentations. Respondents tend to be distracted by many irrelevant questions. The questions presuppose a shared definition of LF by communities and the designers or implementers of the survey tool. Unsurprisingly, Misima Island respondents had difficulty relating to the bio-medical interpretation of LF that underpinned the question outline [forthcoming paper].

In the second stage of our adaptation, we used the findings from our PNG experience to inform the redesign of the tool for use on the Federated States of Micronesia. Before data collection began, key theme areas were identified. On the basis of these themes, open-ended questions were designed to prompt discussion around these issues [11]. Before introducing the issue of LF, local health priorities were explored and the health issues considered important from a local perspective were identified. The question outline then described various LF symptoms and prompted discussion regarding the presence of such manifestations in the area, local names, and local approaches to treating the disease. Following this discussion, the question outline introduced the topic of the disease known as LF, probing whether local people considered it the same disease, which then led into discussion concerning the formal health system's approach to and treatment of the disease.

On the islands of Satawal, Weno and Chuuk in FSM, the local chief, a local health worker, a religious leader, a women's group leader, and a teacher were interviewed. In addition to these specific individuals, six randomly selected (3 male and 3 female) village members were consulted and asked to identify the figures in the community that they perceived to be key informants. Emphasising content over structure, the conversational style of the interview process proved very effective in eliciting insightful understandings.

### Realistic expectations of socio-cultural research

In gathering socio-cultural information, researchers must begin from the perspective of the people with whom they are trying to establish a meaningful dialogue. Attempts to administer qualitative-style questions in the manner of a quantitative structured study design will not yield valuable results. If we assume that the people we are interviewing share our biomedical interpretation of disease, we will engage in exchanges that do not improve our understanding of prevailing perceptions or the impact of LF on affected people's lives. Flawed information or understanding leads us to establish sub-optimal programmes [3].

Valid qualitative research approaches can provide immensely useful data that allows us to design educational and interventional programmes in partnership with affected communities that will ensure high coverage, community support and sustainable control benefits [12,13]. Even an imperfect protocol allowed identification of a number of important issues related to MDA delivery in PNG that have implications for future planning of LF control in that country. Perhaps most importantly, we found that, while the programme depended heavily on the authority of village elders, the younger generation were beginning to resist continued compliance with MDA-style programmes. As the effects of LF were rarely seen, some younger people expressed the belief that the disease was no longer a threat and were not convinced that they would participate in future MDA campaigns. Despite the attempts that had been made to inform people of the nature of the disease, misinformation and misunderstanding were commonly expressed

In FSM, refined data collection techniques permitted more holistic understandings of LF and its affect on the health of the community. An in-depth snapshot of local understanding of basic health beliefs and health service provision in the three communities was established, while understandings and behaviours relating to the occurrence of LF were successfully probed. Although the majority of islanders knew or had heard of someone with elephantiasis or hydrocoele, there was very limited understanding of the cause or transmission cycle of LF. Confusion existed regarding the prevention and treatment of LF, and health staff themselves were insufficiently informed to educate people. Individuals from the outer islands generally appeared to delay until they were suffering from complications before seeking help from the health services on the larger island. Fever attacks and lymphatic tissue inflammation consistent with acute adenolymphangitis was widely reported, and these symptoms were frequently attributed to super-natural causes [8].

These basic findings, although requiring more in-depth study, provide an important framework from which to begin to plan the information campaign necessary to assist the community in interpreting LF transmission and maximally benefiting from appropriate care-seeking.

## Summary

The increasing interest in socio-cultural LF research presents a unique opportunity for coupling socio-cultural and bio-medical understandings of LF, however, the limited tradition in the former is striking. Although, it would be preferable to conduct long-term ethnographic studies in populations affected by LF, the firm commitment to a time-bound elimination programme demands rapid appraisal approaches. The initial insights gleaned from in-depth interviews and focus group discussions with key informants regarding their beliefs and practices related to LF and its impact on local communities clearly indicates the immense value of applying this knowledge to further enhancing the efforts of communities and control programmes. The analysis of socio-cultural data, supported and triangulated with concurrent parasitological, epidemiological and entomological data provides a very valuable repository of information for adapting and refining control efforts that are both responsive to community needs and epidemiological realities.

## Competing interests

The author(s) declare that they have no competing interests.

## Authors' contributions

All authors contributed to the preparation of this manuscript, and have read and approved the final manuscript. SW coordinated the studies mentioned.

SW and DD designed and analysed these studies; JC collected the data in the field and assisted in analysis; BS, JPC, PL and WM assisted in study design and logistical planning of the studies.
